# Scanning with Laser Beam over the TiO_2_ Nanotubes Covered with Thin Chromium Layers towards the Activation of the Material under the Visible Light

**DOI:** 10.3390/ma16072572

**Published:** 2023-03-23

**Authors:** Katarzyna Grochowska, Łukasz Haryński, Jakub Karczewski, Kacper Jurak, Katarzyna Siuzdak

**Affiliations:** 1 Centre for Plasma and Laser Engineering, Institute of Fluid-Flow Machinery, Polish Academy of Sciences, Fiszera 14 Street, 80-231 Gdańsk, Poland; 2 Faculty of Applied Physics and Mathematics, Institute of Nanotechnology and Materials Engineering, Gdańsk University of Technology, Narutowicza 11/12 Street, 80-233 Gdańsk, Poland; 3 Department of Biomedical Engineering, Faculty of Electronics, Telecommunications and Informatics, Gdańsk University of Technology, Narutowicza 11/12 Street, 80-233 Gdańsk, Poland

**Keywords:** laser treatment, TiO_2_ nanotubes, chromium oxide, magnetron deposition, photoelectrochemical properties

## Abstract

This work presents pulsed UV laser treatment (355 nm, 2 Hz) of TiO_2_ nanotubes decorated with chromium oxides. The modification was performed in a system equipped with a beam homogenizer, and during the irradiation, the samples were mounted onto the moving motorized table. In such a system, both precisely selected areas and any large area of the sample can be modified. Photoelectrochemical tests revealed photoresponse of laser-treated samples up to 1.37- and 18-fold under the illumination with ultraviolet-visible and visible light, respectively, in comparison to bare titania. Optimal beam energy fluence regarding sample photoresponse has been established. Scanning electron microscopy images, X-ray diffraction patterns, along with Raman and X-ray photoelectron spectra, suggest that the enhanced photoresponse results from changes solely induced in the layer of chromium oxides. It is believed that the results of the present work will contribute to a wider interest in laser modification of semiconductors exhibiting improved photoelectrochemical activity.

## 1. Introduction

The shortage of natural resources due to the burning of fossil fuels has become a global concern over the past decades [1,2,3]. Moreover, rapid industrial development as well as population increment caused the raising of organic and inorganic contaminants that can influence not only the environment but also human health [4,5]. Taking the above into account, as well as the infinite energy demand in current society, new and green technologies are required to overcome these problems. The common sources of renewable energy are, e.g., solar, wind, geothermal, and ocean energies. Among those, solar energy is not only the most abundant one but is also constantly being replenished. Moreover, the usage of photoelectrochemical devices employing semiconductor electrodes is already widely recognized as an efficient and eco-friendly alternative to explore sustainable energy sources [6,7,8,9].

The reason why semiconductors are used as electrodes for electrochemical processes lies in the energy specificity of semiconductor reactions [10]. It is known that the change in potential of such an electrode may lead to changes in the number of charge carriers [11]. Therefore, various kinds of semiconductors such as Fe_2_O_3_ [12], WO_3_ [13], BiVO_4_ [14], or TiO_2_ [15] are being investigated. Nonetheless, due to the great chemical stability, low toxicity, and cheap price, titanium dioxide is the most favored candidate. In particular, titanium dioxide in the form of nanotubes has been intensively studied due to the excellent electron transfer, easily controllable architecture, and high specific surface area [16]. However, one cannot forget about the drawbacks that hamper the application of pure titania. Most of all, titania exhibits wide band gap of 3.2 eV and 3.0 eV for anatase and rutile phases, respectively [17], meaning that it is not matching to the visible light region, and UV light is mainly absorbed by such material [18]. Another limitation is the fast recombination of electron–hole pairs formed by the titania [19]. According to the literature survey, the modification of titania with non-metals, metals, and metal oxides causes not only the reduction of the energy band gap contributing to the visible light absorption, but also improves the separation of charge carriers leading to the inhibition of electron–hole recombination [15,20].

As transition metals are earth abundant, they stand as a great alternative for noble metals such as gold, platinum, or palladium. Especially, taking into account the deficient resources and high prices of noble metals that make their usage on the large scale pointless. Going further, the main advantages of metal oxides are stability, low cost, and relatively easy preparation routes such as, e.g., electrochemical deposition, photoreduction, wet impregnation, or magnetron deposition combined with annealing [15,21]. In our previous work, we studied titanium dioxide nanotubes decorated with chromium, molybdenum, and tungsten oxides [22]. These oxides are in general regarded as wide band gap semiconductors [23,24,25]. Moreover, chromium oxide exhibits p-type semiconducting properties [26], whereas molybdenum and tungsten oxides exhibit n-type [27,28]. We demonstrated that the deposition of chromium oxides onto the top of the TiO_2_ nanotubes (TNT) increased their photoresponse. Such behavior can be attributed to the intraband states provided by chromium oxides and the change in electronic properties; namely, the additional direct (allowed) pathway for photon absorption. Meanwhile, the deposition of molybdenum and tungsten oxides resulted in decreased photoresponse of the studied material. It was concluded that optical transition occurring in these electrodes has a forbidden nature.

In this work, we extended our studies of titania nanotubes decorated with chromium oxides [22] by exploring the possibility of UV laser modification. It should be noted in here that previously we also examined laser-treated TiO_2_ nanotubes with deposited thin films of chromium [29]. The 532 nm wavelength of Nd: YAG laser was used, though, the photoresponse of prepared material under UV-vis illumination was decreased after modification in comparison to pure titania, while for visible light it increased. Meanwhile surface modification of bare TiO_2_ nanotubes via pulsed UV laser treatment can improve their photoresponse if processing parameters are satisfied [30,31]. Surface melting (the top of the nanotubes) [30,31], degradation of crystal structure [30,31], optical band gap decrement [30], and change in electronic properties, including the increase in donor density [30,31] and positive shift of flat band potential [30], may accompany the enhanced photoresponse. Nonetheless, the general concept of laser treatment is to manipulate crystal structure, which contributes to the emergence of new additional mid-gap states. These states provide additional carriers which narrow optical band gap and enhance sample photoresponse. Therefore, one can expect the synergistic effect of chromium oxide decoration and UV laser processing of titania material.

Indeed, in here we report on optimized pulsed UV laser processing of TiO_2_ nanotubes decorated with aggregates of chromium oxides, resulting in the superior photoresponse. The effect of laser modification on morphology, crystallinity, surface chemical composition, and optical properties was studied by scanning electron microscopy (SEM), X-ray diffraction (XRD), X-ray photoelectron spectroscopy (XPS), and ultraviolet-visible (UV-vis) spectroscopy, respectively. Electrochemical and photoelectrochemical properties of materials were tested by means of cyclic and linear voltammetry; the latter being investigated under UV-vis and vis illumination. Electronic properties were evaluated by analysis of Mott–Schottky plots. Thanks to the use of a beam homogenizer ensuring a square laser spot and motorized table on which the samples were mounted, both precisely selected areas as well as any large area of the sample can be modified. Therefore, such an approach can be further scaled up from the laboratory to a commercial level.

## 2. Methods

TiO_2_ nanotubes were prepared via an anodization process of Ti foil (99.7%, Strem, Newburyport, MA, USA) in a two-electrode system. First of all, titanium foils of 2 × 3 cm^2^ pieces were cleaned in an ultrasonic bath in acetone (Chempur, Piekary Śląskie, Poland, p.a. grade), ethanol (Chempur, p.a. grade), and deionized water (HYDROLAB, Straszyn, Poland, 0.05 µS) for 10 min in each solvent, followed by drying in a stream of cold air (Air Liquide, Paris, France, ALPHAGAZ™ 1 Air). Afterwards, Ti foil served as an anode while Pt mesh as a cathode. The electrolyte consisted of 0.5 g NH_4_F (Chempur, p.a. grade), 7.5 mL deionized H_2_O, and 42.5 mL ethylene glycol (Chempur, p.a. grade). 50 mL of electrolyte was used for the anodization process and the distance between the electrodes was 2 cm. The temperature was controlled by a thermostat (Julabo F-12, Seelbach, Germany) and kept at 23 °C. The voltage of 40 V was maintained for 15 min while the voltage ramp-up and ramp-down rates were set to 6 V min^−1^. As-anodized samples were rinsed with ethanol and were dried in the air freely. Finally, amorphous TiO_2_ nanotubes were annealed in a tubular furnace (Nabertherm, Lilienthal, Germany, 120/1000/12-P330) at 450 °C in the air for 2 h (ramp-up rate: 2 °C min^−1^) to ensure the crystalline phase. The samples were then allowed to cool down freely [22].

In the next step, TiO_2_ nanotubes were covered with 15 nm of chromium film by means of a magnetron sputtering system (Quorum, Q150T S, target: TK8845 Cr, Quorum Technologies, Lewes, UK) equipped with quartz crystal microbalance. Prior the deposition, the target was cleaned by applying 150 mA with the shutter closed. For deposition, the pressure and current were established to 3 × 10^−3^ mBar and 120 mA, respectively. Afterwards, the samples were thermally treated in the tubular furnace to both crystallize and oxidize the deposited material. The processing parameters were the same as in the case of crystallization of titania nanotubes.

Finally, laser modifications were performed using Nd: YAG pulsed laser (Quantel, Lannion, France, Q-850) operated at 355 nm with 2 Hz repetition rate and 6 ns pulse duration. Beam homogenizer was utilized to ensure the uniform intensity spot of square shape. Moreover, the samples were mounted on the precise programmable motorized table (SmarAct, Oldenburg, Germany). The constant speed of the table of 4 mm min^−1^ was maintained during processing. The energy fluences were set to 10, 20, 30, 40, and 50 mJ cm^−2^, while the pressure to 5 × 10^−5^ mbar [30]. The electrode surface was scanned twice (forward and backwards) with a laser beam. The laser spot that reaches the sample is of square shape with 2.3 mm side. It means that to process the 2 × 3 cm^2^ sample the position of laser beam after each treated row of 2.3 mm width should be changed. Every row of the sample is treated with the same energy fluence, and the number of rows is limited only with the size of the sample, enabling this technique to be easily adapted for large-area sample modifications.

The samples’ morphology was investigated using scanning electron microscope technique (FEI, Waltham, MA, USA, Quanta FEG 250) with field emission and ET secondary electron detector. The voltage was kept at 10 kV.

High-resolution X-ray photoelectron spectroscopy analyses were carried out in the core-level binding energy of Ti *2p*, O *1s*, and Cr *2p* to evaluate the surface chemical states of the studied samples. Escalab 250 Xi was used, operating with Al Kα X-ray source, 250 μm spot diameter. The pass energy was 20 eV. Low-energy electron and Ar^+^ ions flow was used for charge compensation, with final x-axis calibration at C *1s* (284.8 eV) adventitious carbon.

The structural analysis was performed by means of X-ray diffractometer (Bruker D2 Phaser 2nd generation, Billerica, MA, USA) using CuKa radiation and a LynxEye XE-T detector at room temperature and Raman spectrometer (InVia, Renishaw, Wotton-under-Edge, Gloucestershire, UK) with laser excitation at 514 nm and power of 2.5 mW.

Optical properties of prepared materials were tested using Lambda 35 UV-Vis Spectrometer in the range of 200–900 nm. The scanning speed was set to 120 nm min^−1^. Band gap energy values were determined on the basis of the Kubelka–Munk function [32].

Bare and the set of modified titania were characterized as electrode materials using different electrochemical techniques. Autolab PGSTAT302N potentiostat–galvanostat system (Methrom, Herisau, Switzerland) was used to control the electrode potential and record the current or impedance data depending on the selected technique. The measurements were carried out in three electrode arrangements, where Pt mesh acted as the counter electrode, Ag/AgCl/0.1 M KCl served as the reference electrode, and the obtained material was treated as the working electrode. The electrolyte was 0.5 M Na_2_SO_4_ with pH = 6.8 deaerated with argon (5.0, Air Liquide, ALPHAGAZ™) for 40 min before measurements. During electrochemical tests, the argon flow was maintained above the solution. Cyclic voltammetry (CV) was carried out in the range from +1.0 V to −1.0 V, with the scanning speed of 50 mV s^−1^. The cyclic voltammetry curves were recorded several times to verify the stability of the electrode material in contact with an electrolyte within the investigated potential range.

Linear sweep voltammetry (LV) measurements were performed in dark and under UV-vis and visible light illumination at 10 mV s^−1^ in the potential range of −0.2–+1.2 V. As a light source, an xenon lamp (Osram XBO 150, Munich, Germany) equipped with an AM 1.5 filter was used. In order to illuminate electrode material with the visible light, an optical filter (420GG Schott) was mounted in the photoelectrochemical setup enabling the cutting off of the wavelengths below 420 nm.

The Mott–Schottky plot was constructed on the basis of the impedance measurements carried out at a constant frequency of 1 kHz, in the potential range from +0.6 V to −0.4 V vs. Ag/AgCl/0.1 M KCl. Before recording data for each potential, the electrode was polarized for 60 s. The donor densities were calculated according to the Mott–Schottky space charge capacitance from the following equation:

(1) $$N_{d}=\left(\frac{2}{\varepsilon_{0}\varepsilon e}\right)[\frac{d\left(\frac{1}{C_{\mathrm{SC}}^{2}}\right)}{\mathrm{dE}}{]}^{-1}$$

where ε is the dielectric constant of TiO_2_, ε_0_ is the vacuum permittivity, e stays for the electron charge, and E is the applied potential; in calculations ε_0_ = 8.85 × 10^−12^ F/m, ε = 38 for anatase-TiO_2_ and e = 1.602 × 10^−19^ C were used [33].

## 3. Results and Discussion

**Figure 1:**
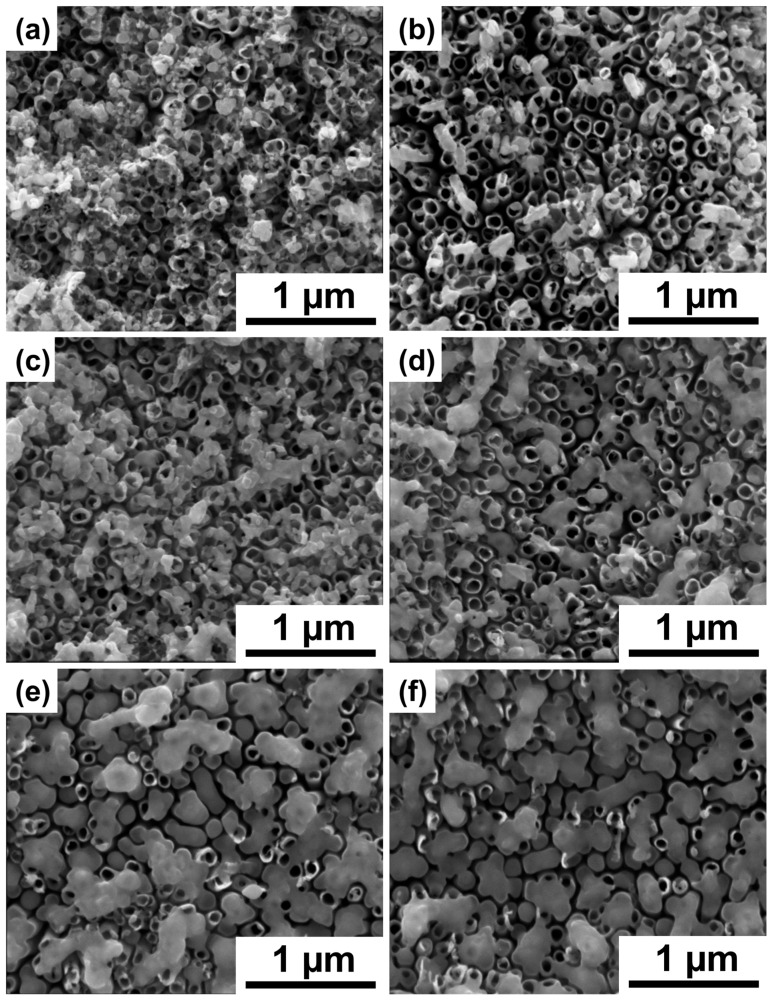
SEM images of the TiO_2_ nanotubes decorated with chromium oxides (**a**) before and (**b**–**f**) after laser treatment with (**b**) 10 mJ cm^−2^, (**c**) 20 mJ cm^−2^, (**d**) 30 mJ cm^−2^, (**e**) 40 mJ cm^−2^, and (**f**) 50 mJ cm^−2^ fluence.

SEM images of the pristine TiO_2_ nanotubes can be found in our previous works [22]. Nonetheless, the length of the nanotubes is of 1.4 ± 0.1 µm, while their internal diameter and wall thickness of 90 ± 8 nm and 13 ± 3 nm, respectively. After deposition, the chromium species are located at the top part of nanotubes in the form of aggregates—see Figure 1a, which is in agreement with our previous reports [22]. SEM images of laser-processed samples are show in Figure 1b–f. It can be easily observed that the lowest laser fluence, namely 10 mJ cm^−2^, did not influence the morphology of the sample in a significant way. However, increasing the laser fluence results in the partial melting of the deposited layer on the top of the nanotubes, and the area of melted area increases with the fluence up to 50 mJ cm^−2^. Similar behavior was previously reported for the bare titania nanotubes [30]; the melted layer can partially or fully cover the nanotube mouths leading to the decrease in surface area. Nonetheless, underneath the melted layer, the architecture of nanotubes remained intact (see Figure S1 in the Supplementary Materials) and the length of TNT was preserved regardless of the parameters of laser processing.

**Figure 2:**
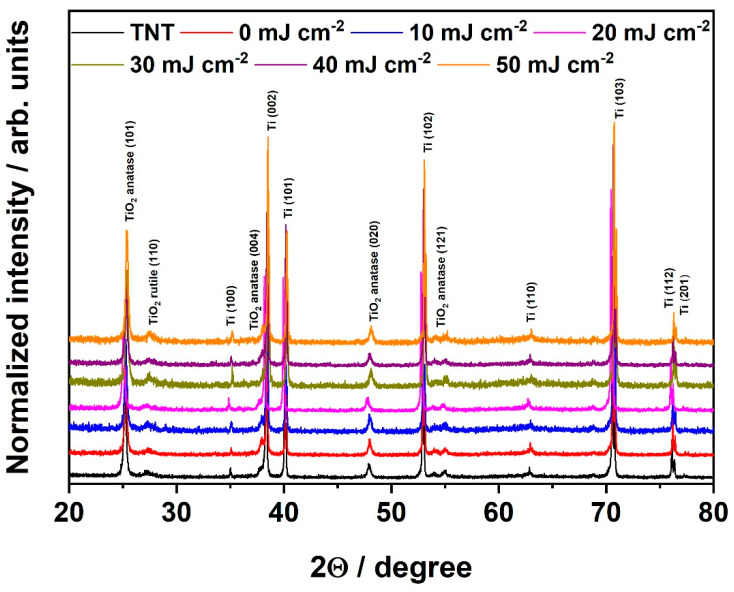
XRD patterns of the TiO_2_ nanotubes decorated with chromium oxides before (0 mJ cm^−2^) and after laser treatment (10–50 mJ cm^−2^).

The structural properties of obtained materials were investigated by means of the XRD technique, and collected XRD patterns confirm the crystallinity of all prepared electrodes. Particular peak positions were identified according to the [ICDD PDF-4+] and are indicated in Figure 2. The anatase is a dominant phase; however, trace rutile peaks can be easily observed. The material phase composition was calculated using the given formulas [34]:

(2) $$W_{A}=\frac{k_{A}A_{A}}{\left(k_{A}A_{A}+A_{R}\right)}$$

(3) $$W_{R}=\frac{A_{R}}{\left(k_{A}A_{A}+A_{R}\right)}$$

where W_A_ and W_R_ are weight fractions of anatase and rutile, A_A_ and A_R_ are integrated intensities of anatase and rutile, and k_A_ is equal to 0.886. Anatase (101) (2Θ = 25.21°) and rutile (110) (2Θ = 27.22°) peaks were analyzed to assess the anatase–rutile fraction.

The reference sample, namely pure TiO_2_ nanotubes, is composed of ca. 88% of anatase and ca. 11% of rutile. Further chromium deposition, thermal annealing in tubular furnace, and laser processing do not influence on the materials composition as well as on the intensity of peaks in a significant way. It can therefore be concluded that the laser beam fluence was not high enough to degrade the crystal structure of the samples [35,36]. It should be also mentioned in here that the diffraction signals of titanium arise from the titanium foil on which nanotubes were grown.

As it comes to material decorated with chromium species, no additional peaks originating from chromium oxide presence are visible in XRD patterns. This can be justified by the low amount of the deposited species which is below the detection limit of XRD instruments. This is in agreement with our previous work [37], in which the XRD patterns of the TiO_2_ nanotubes, decorated with spherical nickel nanoparticles with an average size of 34 ± 10 nm, revealed no peaks corresponding to nickel species.

**Figure 3:**
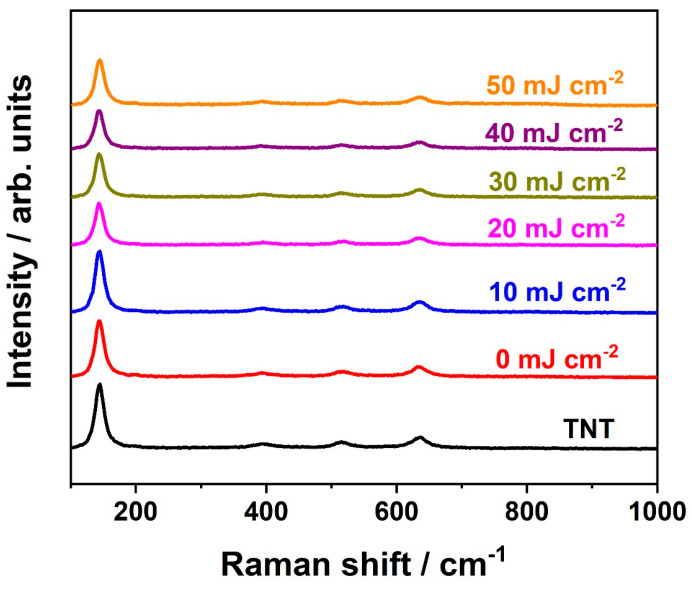
Raman spectra of the TiO_2_ nanotubes decorated with chromium oxides before (0 mJ cm^−2^) and after laser treatment (10–50 mJ cm^−2^).

Further analysis of structural properties was conducted via Raman spectroscopy, and the spectra collected for all the samples are given in Figure 3. Signals registered at 144, 197, 395, 515, and 636 cm^−1^ correspond to E_g(1)_, E_g(2)_, B_1g_, A_1g_, and E_g(3)_ active anatase modes, respectively [38]. On contrary to the X-ray diffractograms, no bands assigned to rutile are observed. This may be explained by the fact that rutile phase is formed underneath the nanotubes, i.e., at the metal/oxide interface [39] and the small amount of this phase. Moreover, the position of the main anatase peak at 144 cm^−1^ remained unchanged regardless of the applied energy fluence used to modify electrode material (Figure S2). It should be mentioned that the change in position was previously observed for pristine laser-treated titania nanotubes [30,40]. Nonetheless, similar behavior, namely no peak shift due to the laser treatment, was also reported for thin gold layers deposited onto TNT [40]. Typically, shift of the 144 cm^−1^ Raman mode as well as its broadening is associated with creation of oxygen vacancies [41,42]. Therefore, obtained results indicate that the additional chromium layer may protect the electrode material from the oxygen vacancies formation which was also assumed in the case of gold film [40]. Nonetheless, it can be also observed that the intensity of the main anatase band varies with different values of energy fluence (Figure S2). It may be caused by the lower degree of crystallinity of laser-treated samples due to the non-homogenous laser irradiation in terms of pulse energy [36] or changes in sample morphology [40]. However, the XRD results do not confirm the former explanation. Therefore, we anticipate that it is attributed to the geometry change—as can be observed in Figure 1.

**Figure 4:**
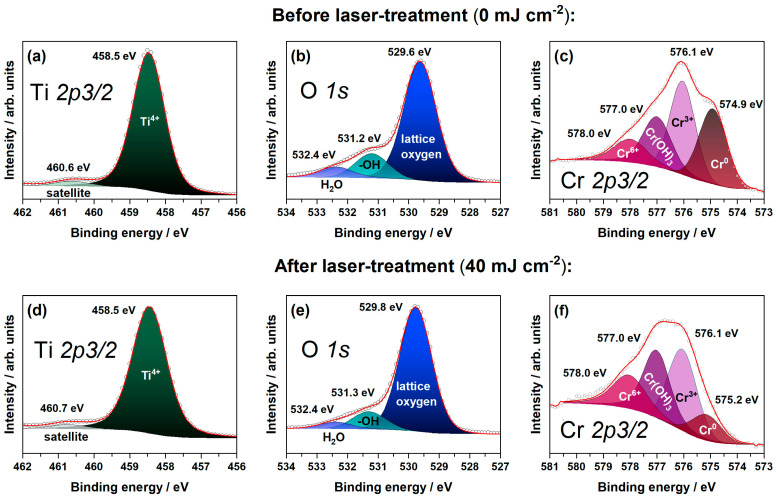
XPS spectra of the (**a**,**d**) Ti *2p*, (**b**,**e**) O *1s*, and (**c**,**f**) Cr *2p* regions registered for the TiO_2_ nanotubes decorated with chromium oxides (**a**–**c**) before and (**d**–**f**) after laser-treatment with 40 mJ cm^−2^.

The surface chemical compositions of the TiO_2_ nanotubes decorated with chromium layer before (Figure 4a–c) and after laser treatment (Figure 4d–f) were investigated by means of the XPS technique. A sample treated with 40 mJ cm^−2^ was selected as a representative one on the basis of electrochemical measurements described later on. The XPS results corresponding to the binding energies for Ti *2p_3/2_*, O *1s* and Cr *2p_3/2_* were analyzed. For laser-untreated electrode material, the high resolution scan of Ti *2p_3/2_* indicates the presence of only Ti^4+^ in the TiO_2_ crystal lattice (peak at 458.5 eV) [43,44]. The signal at 460.6 eV is recognized as a satellite based on its intensity and position from the Ti^4+^ peak [22]. Additionally, no peaks corresponding to the Ti^3+^ are observed. The high-resolution scan of O *1s* can be deconvoluted into three peaks assigned to lattice oxygen, OH, and H_2_O bonds at 529.6 eV, 531.2 eV, and 532.4 eV, respectively [45,46]. For Cr *2p3/2*, the spectrum deconvolution exhibits that peaks at 574.9 eV, 576.1 eV, 577.0 eV, and 578.0 eV correspond to metallic chromium, Cr^3+^, chromium-hydroxide, and Cr^6+^, respectively [47,48,49,50]. The presence of metallic chromium indicates that the sputtered chromium layer did not undergo full oxidation after thermal annealing in the tubular furnace. Additionally, 30% of the total chromium content in the sample can be ascribed to a metallic state; 34%—to Cr^3+^, 23%—to chromium in chromium hydroxide, and 13%—to Cr^6+^.

After laser treatment, no changes in chemical nature of titania are observed as the position of the peak corresponding to Ti^4+^ in the Ti *2p3/2* region remains intact. Also, no Ti^3+^ has been identified. Therefore, XPS measurements verify the preliminary conclusions derived from XRD and Raman spectra regarding the preservation of crystal structure after laser processing. Furthermore, in O *1s* and Cr *2p3/2* regions, insignificant peak shifts can be noticed—see Figure 4e,f. However, taking into account the area under the peaks, it can be concluded that the amount of lattice oxygen increases, while the decrease of OH species occurs with practically no changes for H_2_O bonds. Meanwhile, the overall content of chromium in the sample is decreased over 2 times in the case of the laser-treated sample in comparison to the untreated one. This may be attributed to the evaporation of the deposited layer [51]. Moreover, the metallic chromium share decreased to 12%, while the Cr^3+^, chromium in chromium-hydroxide, and Cr^6+^ increased to 35%, 32%, and 21%, respectively. It should be mentioned in here that lasers have been applied for oxidation of thin films, including chromium layers [52], and the oxidation is still possible under the vacuum conditions [53]. Additionally, the chromium–oxygen bonds were already present due to the initial thermal treatment in air after chromium deposition onto titania nanotubes; therefore, the structural reordering of the oxide species layer due to the subsequent laser heating cannot be excluded [54].

**Figure 5:**
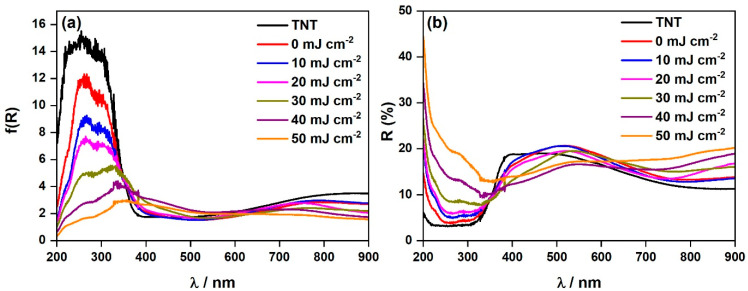
Absorption data of the bare TiO_2_ nanotubes and the laser-treated TiO_2_ nanotubes decorated with chromium oxides (0–50 mJ cm^−2^) displayed as (**a**) Kubelka–Munk function and (**b**) reflectance vs. wavelength.

The results of the reflectance measurements and respective Kubelka–Munk functions are presented in Figure 5. For all the prepared samples, the absorption is primarily limited to the UV part of the spectrum that is typical for titania-based materials [55]. As it comes to pristine titania, it is well-known that the energy band gap values of rutile and anatase are 3.0 eV and 3.2 eV, respectively. For bulk material [56] therefore, the absorption of this material is consistent with the reported wide band gap. The TiO_2_ nanotubes decorated with chromium oxides (0 mJ cm^−2^) exhibit an Urbach tail [57], indicating that the chromium oxides provide intraband gap states. Subsequent laser treatment (10–50 mJ cm^−2^) contributes, however, to a decrease of absorbance (i.e., f(R)) in the UV range. Nonetheless, the absorption edge is redshifted with the increased laser fluence. To access the energy band gap values, the Tauc plots were constructed (Figure S3) and derived values are given in Table S1. For pristine titania nanotubes, indirect (allowed) optical transition was assumed according to the literature [32,58]. The estimated value of 3.01 eV is close to the reported data, nonetheless one cannot forget that it is morphology dependent [59]. In the case of chromium species, for chromium oxide, two different transitions are reported; namely, direct [60] and indirect [23]. Nonetheless, in our previous work we experimentally determined the Tauc exponent that could be assigned to direct (allowed) transition [32]. From Table S1 it is clear that with the increase of laser fluence, the band gap value shifts from 3.14 eV to even 1.68 eV. As no oxygen vacancies were detected by XPS measurements, the changes both in the absorbance and in band gap values are most likely correlated with the differences in sample morphology [61]. This is verified with the reflectance data (Figure 5b) and SEM analysis (Figure 1) as the increase of reflectance with the lowering of the surface area is observed.

**Figure 6:**
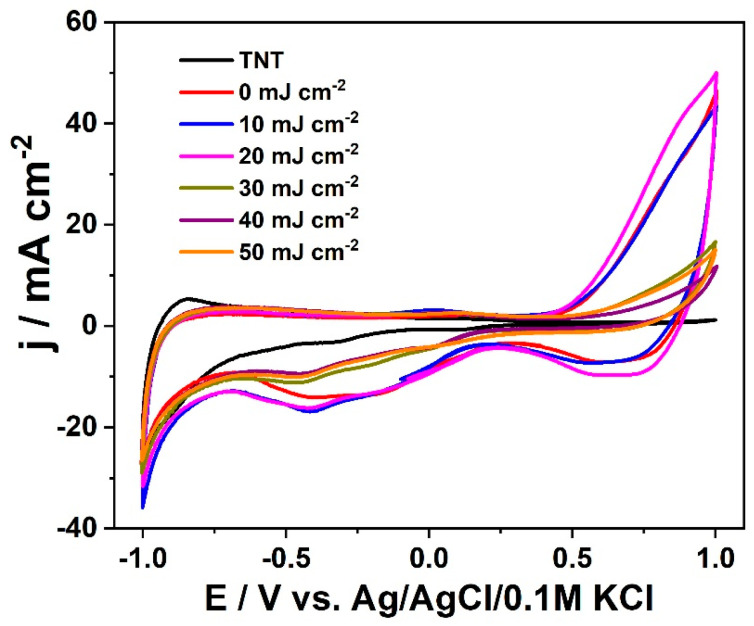
Cyclic voltammetry curves recorded for bare and modified titania recorded with 50 mV s^−1^ in 0.5 M Na_2_SO_4_.

The electrochemical characterization was started with the recording of voltammetry curves in the range from +1.0 to −1.0 V vs. Ag/AgCl/0.1 M KCl that are presented in Figure 6. The working electrode was cycled several times and any current diminishing was not observed, indicating the material stability in neutral electrolyte in the investigated potential range (see Figure S4).

Pure titanium dioxide exhibits the lowest capacitive current densities and no redox reaction especially in the range from −0.7 to +1.0 V [62,63]. The increase of cathodic current observed in the negative range is attributed to the reduction/oxidation of titanium on the surface bonded to the hydroxyl groups [64]. Such activity is many times attributed to doping/de-doping with H^+^ with simultaneous reduction/oxidation of Ti^4+^/Ti^3+^ for TiO_2_ in the form of both nanotubes and single crystal [65,66]. For the electrode modified with chromium oxide, one may observe additional electrochemical activity and the increased current density compared to bare titania in the anodic regime. This feature is well-seen for titania decorated with chromium oxide but without laser treatment and treated with laser beam of low fluence (10 and 20 mJ cm^−2^), and can be ascribed to the oxidation of Cr^3+^ (present in the form of Cr_2_O_3_ as indicated by XPS data) to Cr^6+^. The activity towards water splitting is rather debatable because no arising bubbles on the electrode surface were observed.

Regarding the cathodic branch, we can distinguish two wide reduction bands. The first one with the maximum at +0.65 V can be identified as reduction of Cr^6+^ to Cr^5+^. The other one at −0.42 V could be attributed to the reduction of Cr^5+^ to Cr^3+^ [67]. Unfortunately, literature studies on the titania modified with chromium oxide are poor in terms of detailed characterization using cyclic voltammetry, and the electrochemical investigations are mostly carried out with linear voltammetry in different light conditions or by performing impedance studies. Thus, taking into account data of standard redox potentials [68], for this signal, we also consider another reaction; namely, reduction of Cr^3+^ to Cr^2+^. Once again, we would like to underline that even though the sample was cycled numerous times, each CV curve overlaps, indicating that those reactions are reversible.

**Figure 7:**
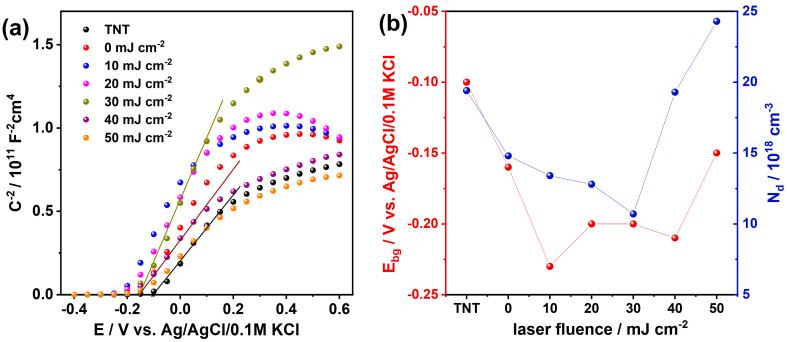
(**a**) Mott–Schottky plot of bare and modified titania in 0.5 M Na_2_SO_4_ solution. (**b**) The change in band gap energy (E_bg_) position and donor density depending on the applied laser fluence. The values of those parameters were determined on the basis of the Mott–Schottky plot given in (**a**).

Then, the impedance measurements were carried out in the range from +0.6 towards −0.4 V vs. Ag/AgCl/0.1M KCl to prepare the Mott–Schottky plot (Figure 7a). The positive slope indicates the characteristic of an n-type semiconductor. The intersection of the tangent of the linear region of the Mott–Schottky plot enables the determining of the position of flat band potential for the surface of fabricated materials.

The flat band potential for pure titania is localized at −0.1 V vs. Ag/AgCl/0.1M KCl, whereas for the laser modified TNT with chromium species the position is negatively shifted, see Figure 7b. When the laser fluence fits the range of 10–40 mJ cm^−2^ the flat band is located at −0.2 V vs. Ag/AgCl/0.1M KCl. In the case of previous work concerning titania modification with chromium oxide, we did not observe any shift [29], but here a cathodic change of 0.1 V is noticed. A similar change was observed by Radecka et al. [69] for Cr-doped titania magnetron sputtered onto the amorphous silica and reported by Li et al. [70] for Zr-doped titania nanotubes modified with urea as well as for iodine-doped titania [71]. In the case of n-type semiconductor, negative shift of flat band potential can indicate that the position of the conduction band is going up and among other factors, can play a role in the charge transfer at the electrode/electrolyte interface.

According to He et al. [72], this negative shift is ascribed to introducing defects levels, thus changing the surface state distribution. However, we should be aware that this change will not only result from the presence of chromium oxide surface species, but can be the effect of laser annealing inducing changes in the morphology and hydroxide groups’ distribution that finally affect the contact area at the electrode/electrolyte interface.

Afterwards, the free-charge carrier concentration was determined on the basis of the slope of the Mott–Schottky plots and using equation (Equation (1)). The change in donor density is depicted in Figure 7b. First, the number of donors decreases reaching the minimum value for fluence equaled to 30 mJ cm^−2^. Then, the donor density increases reaching the maximum for the material annealed with the highest laser fluence and exhibiting the most distorted surface; namely, initially open nanotubes are fully or partially closed. Those changes result both from the changes in the contribution of the oxidation state of Cr in its different species present on TNT surface as well as the decrease of OH species according to XPS investigation.

**Figure 8:**
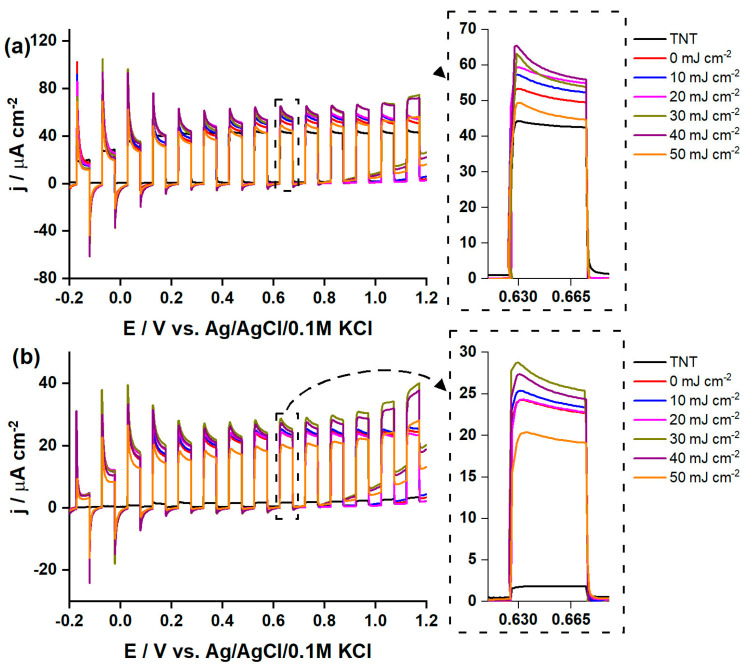
Linear voltammograms registered for the bare and modified titania nanotubes. The LV curves were recorded under chopped (**a**) UV-vis and (**b**) vis irradiation.

Finally, the photoactivity of the fabricated materials was tested. Figure 8a displays linear voltammograms registered under chopped UV-vis illumination of the investigated samples. Note that photocurrent is defined as a difference between current density registered under sample illumination and in dark. In general, all the samples exhibit positive photocurrent along with anodic polarization, indicating n-type semiconducting properties [73]. Current spikes observed at lower potentials, particularly in a range from −0.2 to +0.2 V are related to electron–hole surface recombination during light-off action where these holes are temporarily stored at the photoelectrode/electrolyte interface [74]. Moreover, this can be the result of decrease in band bending [75] due to the cathodic shift of flad band potential, as was confirmed by Mott–Schottky analysis. Bare titania can generate photocurrent of 43 µA cm^−2^ at +0.65 V. Higher photoresponse of about 51 µA cm^−2^ was found for the TiO_2_ nanotubes decorated with Cr oxides but without laser annealing. Subsequent laser treatment with 10–40 mJ cm^−2^ enhanced the photoresponse, and the current density reaches 59 µA cm^−2^. A decrease in photoresponse was found for 50 mJ cm^−2^, indicating that laser fluence higher than 40 mJ cm^−2^ does not ensure greater enhancement of the photoactivity. Additionally, the stability of the material under long-term irradiation was verified and during the whole irradiation period the current was stable and any significant decrease was not observed (Figure S5).

Figure 8b displays linear voltammograms registered under chopped illumination by visible light (λ > 420 nm). Similar character of current spikes was noted as in the case of measurements carried out under UV-vis illumination, suggesting that the same surface recombination processes occur also when the electrode material is exposed to the visible light. As one can expect, this time bare TNT exhibits much lower photocurrent of only ca. 1.5 µA cm^−2^ at +0.65 V due to the wide band gap of titania. When the TiO_2_ nanotubes decorated with Cr oxides (0 mJ cm^−2^) were tested, a huge improvement was recorded, and the sample reaches 23 µA cm^−2^ at +0.65 V vs. Ag/AgCl/0.1 M KCl. It should be mentioned in here that although this sample has wider energy band gap (see Table S1), it also exhibits the Urbach tail (Figure 5) which indicates that the chromium oxides provide intraband gap states. The additional intraband gap states may provide the pathway for electron transfer between semiconductor and the acceptor species, and the excess of electron originating from localized states can affect the surface chemistry of electrode. Therefore, it can be assumed that the intraband gap states can play a major role in visible light response [22]. Subsequent laser modification ensures enhancement of current up to 27 µA cm^−2^ for a beam energy fluence of 30 mJ cm^−2^. However, it should be noted that the current density obtained for the material modified with 40 mJ cm^−2^ is not of much lower value. Thus, it can be stated that the optimum laser fluence fits the range of 30–40 mJ cm^−2^. Such an increase can result from several factors; namely, the decrease of band gap energy, change of the surface morphology that plays the crucial role in contact between the electrode and electrolyte and the charge transfer processes occurring at this interface, and change in the oxidation state of surface chromium oxide species as well as hydroxide groups. Finally, we should also take into account that laser treatment leads to manipulation of band bending that affects the charge transfer, and in this process intraband gap states can also be included.

Combined modification covering the chromium oxides decoration and subsequent laser treatment give a contribution to enhanced photoresponse up to 1.37-fold under radiation, covering the full solar spectrum compared to the bare TiO_2_ nanotubes. Whereas, the laser treatment itself, compared to the unmodified TiO_2_ nanotubes decorated with chromium oxides, provides ca. 20% current density improvement. In the case of visible light, all applied modification routes provide significant enhancement compared to bare titania. Without laser annealing, current density increased over 15 times; whereas, for the laser fluence of 30 mJ cm^−2^, the enhancement factor equals 18. Observed behavior upon both UV-vis and vis irradiation differs from our previous achievements [29], where we worked with the spaced titania nanotubes and no thermal treatment was applied after Cr sputtering. This differences in the fabrication route indicates how initial architecture of nanotubes and the sequence of thermal processing impact the overall photoresponse of the fabricated material decorated with chromium oxide. Moreover, recorded photocurrent improvement upon radiation from the visible range is much higher than obtained for Cr_2_O_3_-loaded titania nanotubes as reported by Ding et al. [76], when potassium dichromate was used as a Cr precursor, and by Radecka et al. [69], who showed that doping with Cr leads to photocurrent lowering due to the acceleration of the recombination process on the point defects. On the other hand, for the titania modified with chromium oxide using chromium nitride as a precursor, Shaislamov et al. [77] reported lower photoactivity under UV-vis light comparing to unmodified titania, while in the visible light conditions, photocurrent of modified electrode was much higher comparing to the bare substrate. A similar case was described by Song et al. [50], who found how annealing atmosphere affects the photoactivity since lack of access to oxygen leads to the formation of oxygen vacancies, affecting in consequence the oxidation state of Cr. In our case, manipulation with laser parameters enables us to change the content of the Cr species while the oxidation state of Ti is preserved. Thus, taking into account former experience [29] and works focused onto the modification with chromium oxide but based on different synthesis routes, the reported increase here of photocurrent for laser-modified titania decorated initially with Cr is a result of the manipulation within the chemical nature of Cr species and surface architecture.

## 4. Conclusions

In this work, a laser-based method of modification of chromium species coated titanium dioxide nanotubes, which leads to enhancement of the photoactivity of the studied materials is presented. Titania nanotubes were prepared via an optimized anodization process followed by thermal annealing to ensure crystallization. After the deposition of Cr layer, additional thermal treatment was applied for both the crystallization and oxidation. Finally, the material underwent annealing by means of UV laser. SEM inspection revealed the changes in the nanotubes’ morphology in terms of partial or full closing of the nanotubes’ tops. Nonetheless, underneath the melted area, the initial architecture of nanotubes remained intact. XRD and Raman measurements confirmed the mainly anatase phase of nanotubes and indicated on no oxygen vacancies present, respectively. The latter was verified with XPS analysis. Moreover, it was proven that the laser treatment influences on the chemical nature of the Cr species, as well as the narrowing of the energy band gap, derived from reflectance spectra. Both Mott–Schottky analysis and LV measurements confirmed the n-type character of prepared electrode materials. For the optimized laser working parameters, the current density of 59 µA cm^−2^ and 27 µA cm^−2^ was registered for samples under UV-vis and vis illumination, respectively. It should be underlined that it gives 1.37-fold and 18 times higher photoresponse, respectively, in comparison to bare titania. Moreover, the usage of beam homogenizer and motorized table ensures the homogenous modification of material of any shape and size, indicating that the proposed route can be easily scaled up from the laboratory to the commercial level.

## Data Availability

The data presented in this study are available on request from the corresponding author. The data are not publicly available due to technical or time limitations.

## Supplementary Materials

The following supporting information can be downloaded at: https://www.mdpi.com/article/10.3390/ma16072572/s1, Figure S1. SEM images (cross-section) of the TiO_2_ nanotubes decorated with chromium oxides (a) before and (b–f) after laser treatment with (b) 10 mJ cm^−2^, (c) 20 mJ cm^−2^, (d) 30 mJ cm^−2^, (e) 40 mJ cm^−2^, (f) 50 mJ cm^−2^ fluence. Figure S2. Raman spectra of the TiO_2_ nanotubes decorated with chromium oxides before (0 mJ cm^−2^) and after laser-treatment (10–50 mJ cm^−2^); the enlarged portion of Figure 3. Figure S3. Tauc plots of (a) the bare TiO_2_ nanotubes and (b) the laser-treated TiO_2_ nanotubes decorated with chromium oxides (0–50 mJ cm^−2^). Figure S4. 20 subsequent CV cycles recorded for the material modified with the laser fluence of 40 mJ cm^−2^. Scan rate was set to 50 mV/s. Figure S5. Chronoamperometry curve recorded under prolonged irradiation of the electrode material modified with 40 mJ cm^−2^ laser fluence. The electrode was polarized at +0.5 V vs. Ag/AgCl/0.1M KCl. Table S1. Optical bandgap values of the bare TiO_2_ nanotubes and the laser-treated TiO_2_ nanotubes decorated with chromium oxides (0–50 mJ cm^−2^).
