# Supplementary material for: Scanning with Laser Beam over the TiO2 Nanotubes Covered with Thin Chromium Layers towards the Activation of the Material under the Visible Light
Source: Materials (Basel). 2023 Mar 23;16(7):2572. doi: 10.3390/ma16072572 (PMC10095246; doi:10.3390/ma16072572)
Supplement: Supplementary file 1 [file materials-16-02572-s001.zip › materials-2268428-supplementary.pdf]

materials

Supplementary Materials

Scanning with laser beam over the TiO_2 nanotubes covered with thin chromium layers towards the activation of the material under the visible light

Katarzyna Grochowska[1], Łukasz Haryński[1], Jakub Karczewski[2], Kacper Jurak[3] and Katarzyna Siuzdak[1]

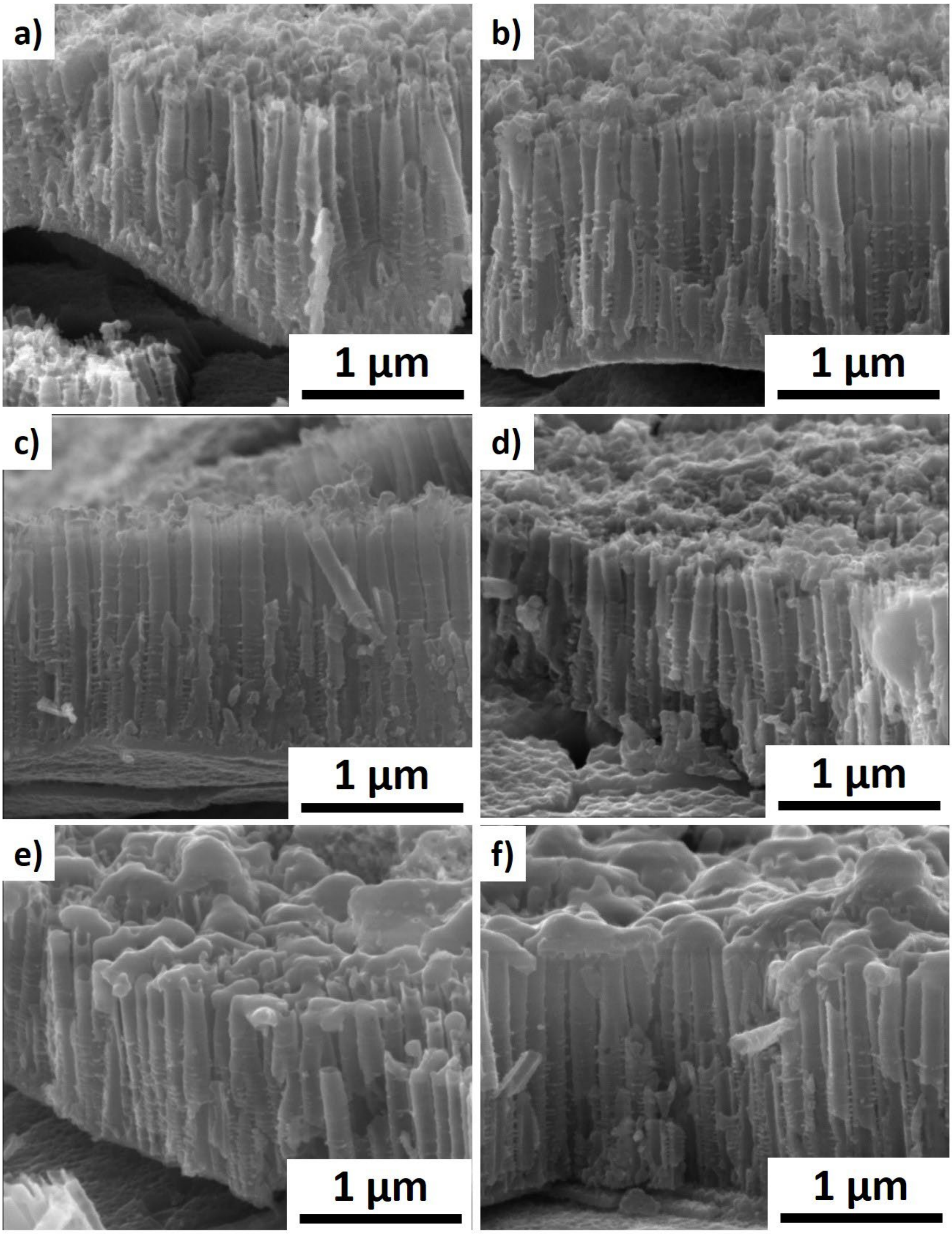

Figure S1. SEM images (cross-section) of the TiO_2 nanotubes decorated with chromium oxides (a) before and (b-f) after laser treatment with (b) 10 mJ cm^{-2}, (c) 20 mJ cm^{-2}, (d) 30 mJ cm^{-2}, (e) 40 mJ cm^{-2}, (f) 50 mJ cm^{-2} fluence.

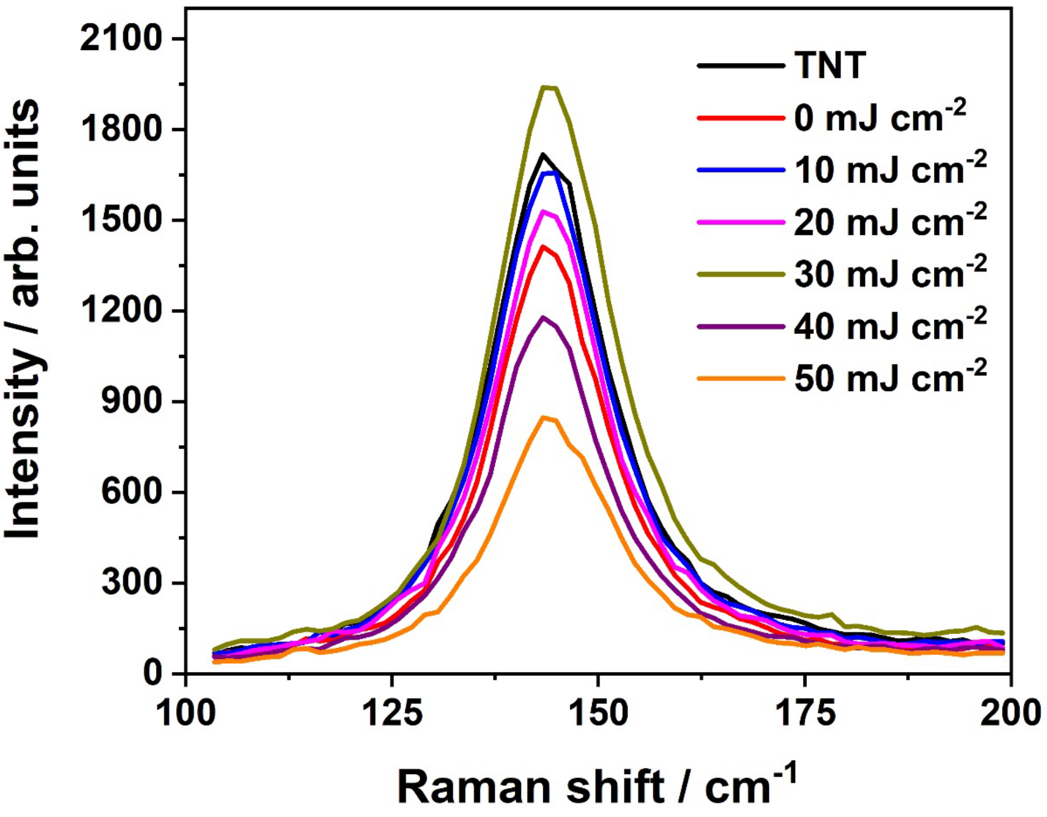

Figure S2. Raman spectra of the TiO_2 nanotubes decorated with chromium oxides before (0 mJ cm^{-2}) and after laser-treatment (10 – 50 mJ cm^{-2}); the enlarged portion of Figure 3.

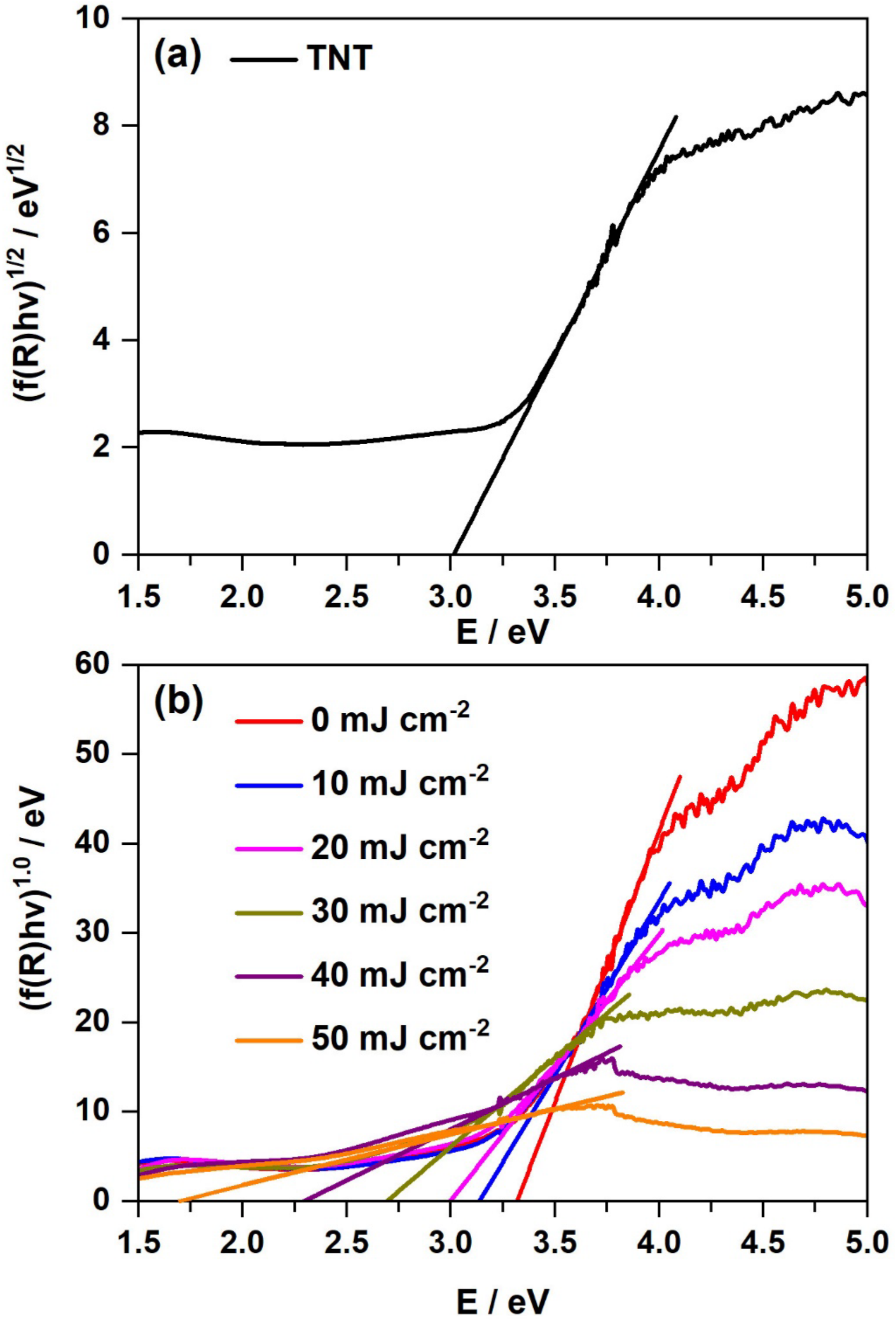

Figure S3. Tauc plots of (a) the bare TiO_2 nanotubes and (b) the laser-treated TiO_2 nanotubes decorated with chromium oxides (0 - 50 mJ cm^{-2}).

Table S1. Optical bandgap values of the bare TiO_2 nanotubes and the laser-treated TiO_2 nanotubes decorated with chromium oxides (0 - 50 mJ cm^{-2}).

Copyright: © 2023 by the authors. Licensee MDPI, Basel, Switzerland. This article is an open access article distributed under the terms and conditions of the Creative Commons Attribution (CC BY) license (https://creativecommons.org/licenses/by/4.0/).

**Sample**	**Bandgap [eV]**
TNT	3.01
0 mJ cm^{-2}	3.32
10 mJ cm^{-2}	3.14
20 mJ cm^{-2}	3.00
30 mJ cm^{-2}	2.70
40 mJ cm^{-2}	2.29
50 mJ cm^{-2}	1.68

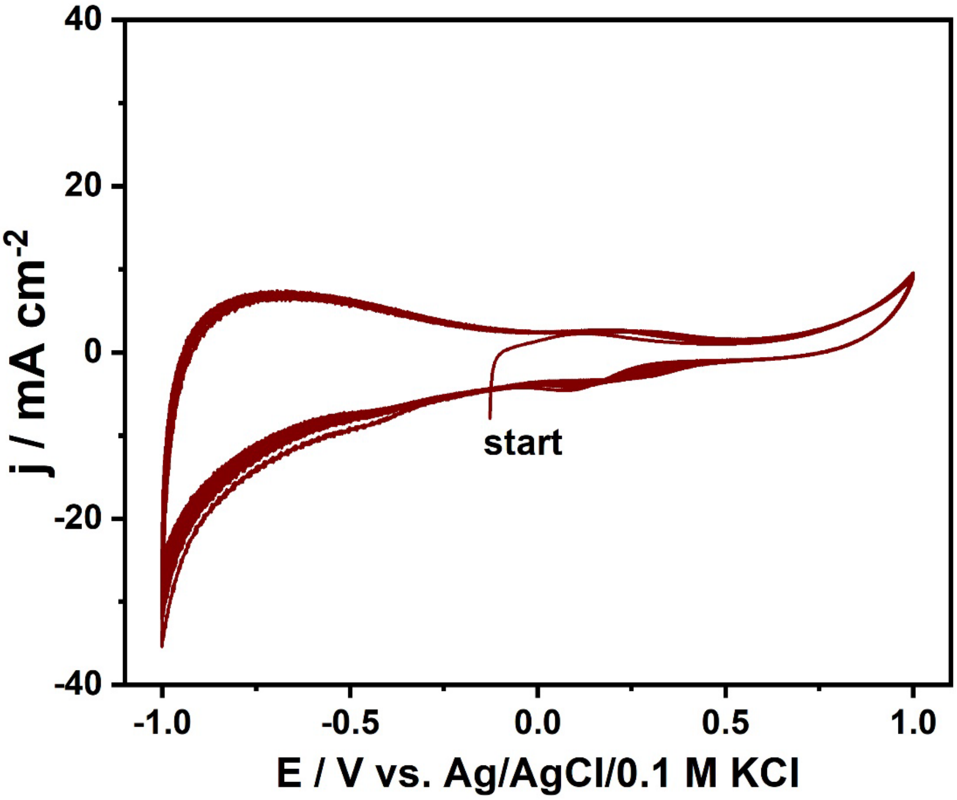

Figure S4. 20 subsequent CV cycles recorded for the material modified with the laser fluence of 40 mJ cm^{-2}. Scan rate was set to 50 mV/s.

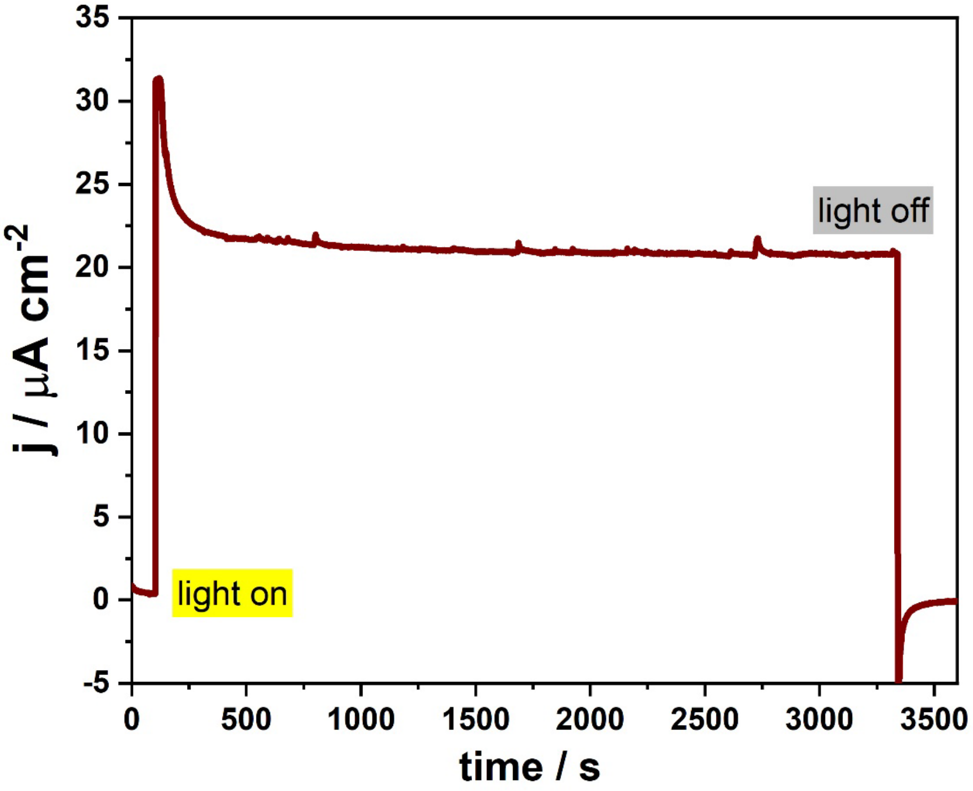

Figure S5. Chronoamperometry curve recorded under prolonged irradiation of the electrode material modified with 40 mJ cm^{-2} laser fluence. The electrode was polarized at +0.5 V vs. Ag/AgCl/0.1M KCl.

When the light starts irradiating the material surface, the recorded current increase rapidly. Then the current falls and reaches the steady-state value as the part of produced electrons and holes recombines. The recombination occurs because holes are accumulated at the electrode/electrolyte interface, while electrons are present in the bulk or those charges are trapped at the surface states. The small cathodic spike is also observed as is in the case of linear voltammogram (see Figure 8 in the main manuscript), so accumulation of holes can be regarded as the main reason of the recombination process.

Disclaimer/Publisher's Note: The statements, opinions and data contained in all publications are solely those of the individual author(s) and contributor(s) and not of MDPI and/or the editor(s). MDPI and/or the editor(s) disclaim responsibility for any injury to people or property resulting from any ideas, methods, instructions or products referred to in the content.
